# Benefits, harms and evidence – reflections from UK primary healthcare

**DOI:** 10.1080/17571472.2017.1384610

**Published:** 2017-10-13

**Authors:** Margaret McCartney

**Affiliations:** General Practitioner and Freelance Writer, Glasgow, UK

**Keywords:** Ethics, evidence, benefit, publication bias, guidelines

## Abstract

In this paper I examine the relationship between benefits, harms and evidence-based medicine in the context of British primary healthcare. First, I will examine: ‘What is a benefit and what is a harm?’ Second, what should we know about where the balance of risk and benefit appear to lie? Third, what should we do with this knowledge, particularly in the context of the biopsychosocial gaze of primary care? I conclude that even perfect knowledge about benefits and harms requires to be translated in the context of the individual patient: it also requires to be interpreted according to what that persons’ wishes are.

**Why this matters to me:**

By reiterating again and again how biases are stacked in favour of recommending treatments and interventions well beyond their rational evidence, my hope is that more honest medicine will result in less but higher value medicine. Stopping doing things that don’t work, or work rarely, or come with an unacceptable burden of side effects or appointments should make room for the pleasure of practicing medicine.

**Key message(s):**

• Even perfect knowledge about benefits and harms requires to be translated in the context of the individual patient: it also requires to be interpreted according to what that persons’ wishes are.
• In the real, messy frontline world of general practice, we will always have uncertainty about where the balance of risk and benefit might lie.

## Introduction

Benefits and harms are wedded like horses and carriages, or bows and arrows. Interventions we (clinicians) offer often give us some of something useful, but rarely include none of something either useless or harmful. In primary care, these qualities are immutably tied together. Naturally, to make decisions as good as possible, we need to know, ‘What is the benefit relative to harm?’

First, I will examine: ‘What is a benefit and what is a harm?’ Second, what should we know about where the balance of risk and benefit appear to lie? Third, what should we do with this knowledge, particularly in the context of the biopsychosocial gaze of primary care?

## What is a benefit and what is a harm?

Many studies have examined the harm of treatments, and death as a comparative measurement in particular. This is an obvious, binary outcome to measure, and is naturally of great importance. It is particularly important in screening studies, where the harm caused by treatment of over-diagnosed conditions can result in death – for example, the surgery for a screening-detected aortic aneurysm incurs a mortality rate – however the aneurysm being operated on may have been destined never to rupture. When we talk of using statins to reduce the risk of future heart disease or stroke, we can compare the expected numbers of heart attacks and strokes in statin-treated against untreated groups.

Then there are other benefits and harms which may be less obvious. For people considering a statin for primary prevention, reasons for not wishing to take it can range from a feeling that one is already consuming too many tablets; that friends or neighbours have had problems; that one would prefer to avoid side effects, visits to the doctor or pharmacist, or, as one person as put it to me ‘I’d rather die of a heart attack than cancer’. Or, in choosing between two anticoagulants, one requiring regular blood tests at a clinic, and the other needing no monitoring, the latter would seem more convenient and therefore obviously preferred. However, some people prefer the former, often because regular contact with a health professional and the social interaction of a clinic visit is viewed as an advantage. Some people may have beliefs about interventions which are changed after a discussion about evidence (for example, the purpose and evidence for preventative vaccination). But many other people have views on interventions which are essentially clashes of competing values and priorities: a guideline whose purpose is to reduce future strokes using tablets is of little value when the person’s stated aim is to avoid tablets.

## What should we know about where the balance of risk and benefit appear to lie?

But what should that evidence which the doctor presents, and which is capable of changing minds, consist of? Judging what is a benefit and what is a harm requires the opinion of the individual to whom the intervention is being offered. Benefits and harms are, in the eye of the doctor, often medico-legal tabulations that must be cited correctly, in purpose to ensure informed consent and legal protection. In practice, our reckonings may be mere inexact gambles, and benefits and harms of the interventions we propose may be more subtle and unquantified. The problem, as we will come to see, is that professionals may present risks and benefits in such ways as to make doing more elaborate or invasive medical interventions seem like the more appealing option. Further, unequivocal harms – such as death or damage from needless surgery- are often framed by using a toxic combination of misleading or incomplete statistics and emotion, making it hard for contemplative citizens to make choices on the basis of clean facts and personal priorities.

In order to place the values held by individuals about risks and benefits, we therefore need to know what outcomes, and in what quantity, we can reasonably expect from medical interventions on offer. These outcomes need to include things that patients would like to know about. In the UK, the General Medical Council (GMC) says that doctors should ‘not make assumptions’ about ‘(a) the information a patient might want or need (b) the clinical or other factors a patient might consider significant, or (c) a patient’s level of knowledge or understanding of what is proposed.’ They go on to say that patients should be given information they want or need about ‘the potential benefits, risks and burdens, and the likelihood of success, for each option’ [1].

This is where the problems really start to accumulate. First, we know that not all the evidence gathered in clinical trials are published, meaning that even the most thorough systematic reviews are capable of reaching misleading conclusions [2,3]. This is not the only bias, but may be ethically challenging. If ethical decisions should be fully informed, selective publication, for example, may prejudice a fully-informed state of knowledge. When data is independently analysed, rather than by the drug company sponsoring, very different conclusions may be reached. Initial trials of antidepressants in teenagers found them to be ‘generally well tolerated and effective’, [4] but independent scrutiny found them to be not effective and was overall harmful [5]. Publication bias means that more trials are published which find benefit than harm, [6]. All of these issues would point towards medical interventions being perhaps not as good in reality as they might appear in research papers.

One might therefore expect some caution when translated into real world practice. Yet when asked about some of these binary outcomes, doctors tend to not only overestimate the cardiovascular risk patients have, [7] and overestimate the benefits of treatment [8]. Doctors overestimate the lifespan of people with type-2 diabetes and overestimate the effect of treatments [9]. No wonder that patients, too, overestimate the benefits of screening tests and underestimate the harms [10]. The biases in how we interpret research continue. If we don’t know about lead time bias we assume that finding more cancer in screened populations is always a good and useful thing. And of course, doctors are very good at using another form of bias, confirmation bias, to justify what we do by seeing only what suits the internal narrative we construct. Naturally, we think that what we offer does more good than harm; we underplay risk, we exaggerate benefits: it is easier all round if we continue to believe in the medicine, and don’t examine the nasty underbelly of unpublished trials, our own and other biases, and bad statistics. But of course, we must.

There are ways to do it better. It is here, in how we reckon with benefit and harm, that we can make useful differences to how we perceive them. When we are told that we can reduce our risk of disease by 50%, a treatment might seem very useful. When we are told that a treatment can reduce our risk of disease from 0.05 to 0.025%, it seems less impressive. This is the difference between relative and absolute risk, and is the cause of recurrent irritability on my behalf when listening to media reports about alleged medical breakthroughs. This – framing – does not necessary lie, but may mislead. There is evidence that presenting the same data in more favourable ways results in people overestimating treatment effects – with doctors and patients getting it wrong in very similar ways [11]. Not surprisingly, presenting data using absolute numbers makes it more likely for doctors to report risk and benefit accurately [12]. Similarly, decision aids- often computer aided – can help people gain greater knowledge about the choices they have – and notably reduce the numbers of people wishing elective surgery [13].

This of course takes time, and rapid general practice appointment systems, with emphasis on throughput rather than the quality of decision-making, makes for a poor starting point.

And even then: what about our unknown unknowns? What about are the things we didn’t ask to begin with: my second question. If we want to help people make good decisions about healthcare options, we need to ensure that we have information about benefits and harms that matter. So, for example, researchers want to do more drug trials; patients are more interested in better information about non-drug treatments [14]. When people with arthritis were asked about their priorities for research, they were not so keen on knowing the impact of treatments on pain, but on fatigue – yet this was not something that researchers were routinely asking about [15]. When patients are not involved in setting up research trials, the benefits and harms we end up knowing about are the ones that doctors identified as being important, not patients. This means that when the findings of the research (which needs patients to take part in, or else it is not done) are made known, they do not give the answers patients wanted. This is wasteful – and essentially unethical.

Or take, for example, the risk of negative psychological effects following screening tests. This was hardly examined in the research evidence leading up to the beginning of many screening programmes in the UK [16]. Who would have thought that the steep rise in early breast ‘cancer’ of the type ‘DICS’ (ductal carcinoma, detected by screening) would be mainly due to overdiagnosis? [17] Overdiagnosis is a harm, because the associated sequelae – surgery, radio or chemotherapy – can never benefit the individual, because the underlying problem was not destined to ever do damage to the person. Yet unless the person has the knowledge that overdiagnosis is possible or likely, the medical experience is likely to be seen as overall beneficial. There may have been painful, time consuming, difficult treatments – but these harms are balanced by the benefit of being alive. If we knew that our life was not under threat, we would be more likely to view the treatments as harmful. Women activists have eloquently reminded doctors that this is not about more information, but better information – and the opportunity to exercise real autonomy [18,19].

## What should we do with this knowledge? A key contribution of the primary healthcare setting

Being able to present benefits and harms accurately and with as little bias as possible is not the end of the story. Medical decision-making is not simply to do with weighing up one against the other, but about the burdens of healthcare, the priority it has, ones’ family, work and interests, philosophy of life, view of death, quality of living, purpose, spirituality, and mental health. Decisions can unfold over time, and a discussion of benefit and harm for most doctors will not involve the exchange of digested numbers needed to treat or harm for a binary yes or no deal. General practice is richer and more subtle than this. At the pinnacle of general practice, it realises a mutual regard and appreciation for values. A study in a journal explained that for 100,000 women being screened for breast cancer, every year from age 40–75 (more frequent than in the UK), 11 deaths due to radiation would be expected. But because they felt more deaths would be stopped through breast cancer, they concluded ‘The risk of radiation-induced breast cancer should not be a deterrent from mammographic screening of women’ [20]. Another study about how strictly to control blood sugar in people with diabetes found that lower sugar levels results in less later complications from the diabetes. However there was a bigger risk of hypoglycemia. Nevertheless the authors wrote that ‘Although we are mindful of the potential for severe injury, we believe that the risk of severe hypoglycemia… is greatly outweighed by the reduction in microvascular and neurologic complications’ [21]. Each of these conclusions is unhelpful because it denies both the values and autonomy of the person at the receiving end. It is not for the researchers to say which of these outcomes is a benefit relative to harms and which intervention it is worthwhile to accept. Even perfect knowledge about benefits and harms requires to be translated in the context of the individual patient: it also requires to be interpreted according to what that persons’ wishes are.

How do we get there? Much work is underway in articulating patient views and values in research and decision aids. This is welcome, but we need more. Clearly, systematic imperatives that push compliance with targets to treat patients rather than offer considerate discussion on options must go. By reiterating again and again how biases are stacked in favour of recommending treatments and interventions well beyond their rational evidence, my hope is that more honest medicine will result in less but higher value medicine. Stopping doing things that don’t work, or work rarely, or come with an unacceptable burden of side effects or appointments should make room for the pleasure of practicing medicine. As I write, in 2016, that joy has been buried under nearly two decades of tedious target culture. By shifting our focus away from the computer, and back onto the person, talking about risks and harms, sharing uncertainties, and talking about priorities – the reason that GPs like me consider that general practice is the best job in the world will surely re-emerge.

## Editor’s note

Readers attending the 2017 RCGP Annual Primary Care Conference may wish to discuss this paper further at a special Fringe Session convened by the RCGP Committee on Medical Ethics ‘Inside GP ethics: Guidelines “and” “of” or “for” Good Clinical Practice’ on *Thursday 12 October 17.15–18.15 in the conference venue*.

The ancient philosopher Plato argued that practicing medicine according to a rulebook was second class medicine (for application to slaves). In today’s practice not following a guideline can seem like acting against a responsible body of medical opinion. In this fringe session, delegates are invited to join the RCGP Committee on Medical Ethics and key invited guests, to discuss the ethics of practicing with guidelines. We will discuss guidelines for decisions about the right treatment, and guidelines about whether to treat. We will discuss whether there are times where a guideline should be followed and when it should not. Is it moral or immoral to incentivise guidelines? We will discuss where guidelines come from in terms of evidence, values and politics, and whether awareness of this matters. Our panel is drawn from Academia, Education and Practice across the UK. We aim in this discussion to promote understanding, to increase awareness of sources of education and support and scholarship, discuss good practice, and air ethical issues generated by this topic in a collegial setting. The discussion will be conducted under Chatham House Rule.

The Speaker Panel will include:

Dr. Andrew Papanikitas – chairing- (University of Oxford, Deputy Chair of COME),

Dr. Paul Myres (RCGP Wales and COME member),

Prof. Paul Thomas (Imperial College London & Editor, London Journal of Primary Care),

Dr. John Spicer (Health Education England, COME Member),

Dr. Carey Lunan (RCGP Scotland, COME Member)

Dr. Benedict Hayhoe (Academic Clinical Lecturer, Imperial College London)

## Disclosures and conflicts of interest

I’m an NHS GP partner, with income partly dependent on Quality and Outcomes Framework points. I’ve written two books and earn from broadcast and written freelance journalism. I’m an unpaid patron of Healthwatch. I make a monthly donation to Keep Our NHS Public. I’m a member of Medact. I’m occasionally paid for time, travel, and accommodation to give talks or have locum fees paid to allow me to give talks but never for any drug or public relations company. I was elected to the national council of the Royal College of General Practitioners in 2013 and am chair of its standing group on overdiagnosis. I was recently appointed onto the RCGP Committee on Medical Ethics. I invested a small amount of money in a social enterprise, Who Made Your Pants?, which went into receivership in 2015.

## Disclosure statement

No potential conflict of interest was reported by the author.
